# Design and Validation of a Handheld Optical Polarization Imager for Preoperative Delineation of Basal Cell Carcinoma

**DOI:** 10.3390/cancers14164049

**Published:** 2022-08-22

**Authors:** Peter R. Jermain, Tyler W. Iorizzo, Mary Maloney, Bassel Mahmoud, Anna N. Yaroslavsky

**Affiliations:** 1 Advanced Biophotonics Laboratory, University of Massachusetts Lowell, Lowell, MA 01854, USA; 2 Department of Radiation Oncology, Massachusetts General Hospital, Boston, MA 02114, USA; 3 Department of Dermatology, University of Massachusetts Memorial Medical Center, Worcester, MA 01605, USA; 4 Department of Dermatology, Massachusetts General Hospital, Boston, MA 02114, USA

**Keywords:** nonmelanoma skin cancer, basal cell carcinoma, optical imaging, polarization

## Abstract

**Simple Summary:**

Skin cancer is the most common malignancy in humans. The goal of this study was to design, implement, and clinically test a novel handheld optical polarization imaging (OPI) system for rapid and noninvasive detection of basal cell carcinoma (BCC) margins. The device is compact, lightweight, and can be operated with minimal training. To validate the handheld imager, 10 subjects with biopsy-confirmed BCC were imaged prior to Mohs surgery. The optical images were processed using a spectral encoding method to increase the accuracy of the tumor boundary delineation. Preoperative margin assessment results from the OPI were compared to the surgeon’s clinical evaluation and to the gold standard of histopathology. Our findings indicate that OPI may be a valuable tool for optimizing surgical treatment of skin cancer.

**Abstract:**

Background: Accurate removal of basal cell carcinoma (BCC) is challenging due to the subtle contrast between cancerous and normal skin. A method aiding with preoperative delineation of BCC margins would be valuable. The aim of this study was to implement and clinically validate a novel handheld optical polarization imaging (OPI) device for rapid, noninvasive, in vivo assessment of skin cancer margins. Methods: The handheld imager was designed, built, and tested. For clinical validation, 10 subjects with biopsy-confirmed BCC were imaged. Presumable cancer margins were marked by the study surgeon. The optical images were spectrally encoded to mitigate the impact of endogenous skin chromophores. The results of OPI and of the surgeon’s preoperative visual assessment were compared to clinical intraoperative histopathology. Results: As compared to the previous prototype, the handheld imager incorporates automated image processing and has 10-times shorter acquisition times. It is twice as light and provides twice as large a field of view. Clinical validation demonstrated that margin assessments using OPI were more accurate than visual assessment by the surgeon. The images were in good correlation with histology in 9 out of 10 cases. Conclusions: Handheld OPI could improve the outcomes of skin cancer treatments without impairing clinical workflows.

## 1. Introduction

Basal cell carcinoma (BCC) is the most common type of cancer [1]. This year in the United States about 3.6 million new BCC lesions will be treated in more than 2 million people [2]. Epidemiological data project increasing incidence of the disease worldwide [3]. At present, several treatment options for BCC including curettage [4], cryotherapy [5], photodynamic therapy (PDT) [6,7,8], radiation therapy [9,10,11], and others are available. However, excisional surgery remains the leading intervention [12,13,14]. During conventional surgical resection, the tumor is removed with a lateral clearance margin (~4–6 mm) of adjacent normal-appearing skin [15]. There is no real- time ability to evaluate whether or not the lesion was completely removed and, consequently, high risk that the residual tumor will remain at the excision site [16]. Mohs micrographic surgery (MMS) technique achieves high cure rates (~99%) using intraoperative evaluation of hematoxylin and eosin (H&E) histopathology for intraoperative margin control [17,18]. This method utilizes horizontal tissue sectioning to enable inspection of the entire surgical margin [19]. However, MMS is expensive, time-consuming, labor-intensive, and not readily available in most dermatology surgery clinics [20,21].

Despite efforts to improve preoperative delineation of skin cancers using epiluminescence microscopy (dermoscopy) [22,23] and optical techniques including integrated reflectance confocal microscopy (RCM)/optical coherence tomography (OCT) systems [24,25,26], initial tumor boundary assessments are still based primarily on visual inspections performed by the surgeon. Outcomes from this approach depend on prior clinical experience and are prone to error because of limited visible contrast between malignant and normal skin [27].

Our group is developing optical polarization imaging (OPI) technology for noninvasive, preoperative detection of nonmelanoma skin cancer (NMSC) margins. In this work, we present a novel handheld OPI system, characterize technical specifications of the device, and provide results from imaging 10 BCC cases to demonstrate its potential for clinical adoption.

## 2. Materials and Methods

### 2.1. Handheld Optical Polarization Imager

**Figure 1:**
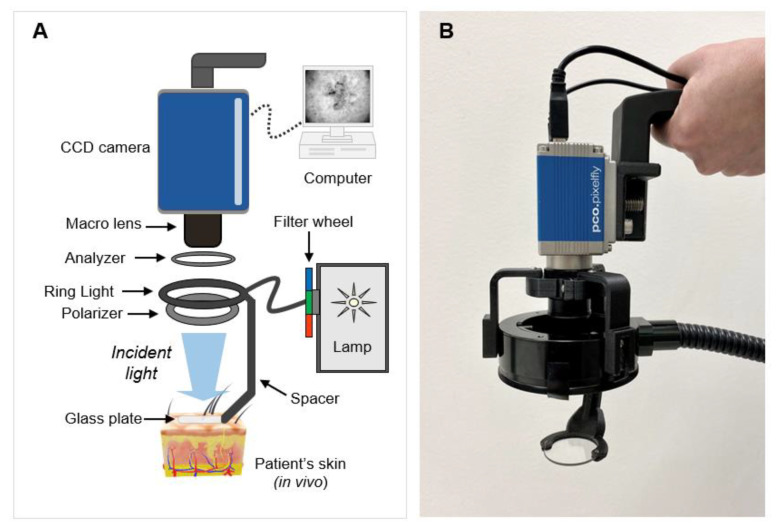
Handheld optical polarization imaging (OPI) system. (**A**) Schematic diagram; (**B**) Photograph of the device.

The optical imaging system is presented in Figure 1. The layout (Figure 1A) included a lamp (Lambda LS, Sutter, Novanto, CA, USA) with a filter wheel to emit narrowband light at 440 nm, 570 nm, or 640 nm (full-width at half maximum (FWHM) = 10 nm). A fiber optic ring light (Edmund Optics, Barrington, IL, USA) equipped with a linear polarizer (Edmund Optics, Barrington, IL, USA) directed light towards the skin. Light reflected from the skin passed through an analyzer and was collected by a 0.3X/F8 macro lens (Rodenstock GmbH, Munich, Germany). A custom, 3D-printed spacer (Rize Inc., Concord, MA, USA) fitted with a 38-mm-diameter glass plate was designed and implemented to ensure the proper imaging distance. Cross-polarized images were acquired with a 14-bit charge-coupled device (CCD) PCO Pixelfly USB camera (PCO Tech, Kelheim, Germany). A 440 nm image visualized the dermal collagen network, a 570 nm image showed vasculature, and a 640 nm image displayed surgical markers on the skin surface used to outline estimated tumor borders. Image acquisition was controlled using MetaMorph software (Molecular Devices, Sunnyvale, CA, USA). The images could be viewed in real time or as single frames on a computer.

### 2.2. Image Processing

An in-house-developed MATLAB code (MathWorks, Natick, Massachusetts) was used to generate spectrally encoded images using the following formula:

*P*_*i,j*_ = ((α × *P*^*440 nm*^_*i,j*_)/*P*^*640 nm*^_*i,j*_) × 1000 (1)

where *P_i,j_*, *P^440 nm^_i,j_*, and *P^640 nm^_i,j_* are pixel values of the spectrally encoded image, 440 image, and 640 nm image, respectively. The constant α is the ratio of mean pixel values in 640 nm and 440 nm images. The spectrally encoded image was scaled by 1000 to optimize display.

Spectral encoding minimized artifacts caused by uneven illumination and by the presence of endogenous skin chromophores (e.g., melanin, oxyhemoglobin, and deoxyhemoglobin). Spectrally encoded images were pseudo-colored using the MATLAB code. Pixels with grayscale values below 20% of the maximum pixel intensity value in the image were set as black and purple colors (representing the tumor), whereas pixels above 20% of the maximum were set as blue/green/yellow/orange/red colors (representing normal skin). Binary images that display cancerous and normal skin as black (lowest 20% of pixel values) and white, respectively, were also generated.

### 2.3. OPI Clinical Evaluation

Clinical performance of the handheld OPI device was evaluated by imaging patients with BCC prior to MMS. The study was performed under a protocol approved by the Institutional Review Board at the University of Massachusetts Medical School (IRB ID H00017121). Enrolled subjects were aged 18 or older, had at least one biopsy-confirmed BCC lesion, and were scheduled to undergo MMS at the University of Massachusetts Medical Center Dermatology Surgery Clinic. Prior to imaging, the surgeon consented the patient and used a sterile purple marker to outline the clinical margins of the excision. The treatment site was cleansed with isopropyl alcohol, and an optically transparent gel (Aftersun Aloe Vera, CVS Health Corporation, Woonsocket, RI, USA) was applied to the lesion to improve light coupling into the skin. Digital photographs of the treatment site were obtained for reference. Optical imaging was performed by researchers from the Advanced Biophotonics Laboratory (ABL) of the University of Massachusetts Lowell. Images were acquired in less than 1 s, while the entire imaging procedure took about 5 min per subject. The study surgeon was blinded to the optical images. Therefore, OPI did not interfere with routine MMS procedures. After imaging, standard MMS was performed based on the surgeon’s initial visual estimate of the tumor size.

### 2.4. Data Analysis

Results from optical imaging were compared to the surgeon’s preoperative visual margin assessment and validated against clinical H&E histopathology. When histopathological analysis revealed no residual tumor at the lateral boundary of the Mohs excision (i.e., negative surgical margins), the preoperative OPI assessment was considered true negative (TN) or false negative (FN) if the spectrally encoded image displayed collagen disruption contained inside or spread outside the surgeon’s markings, respectively. In cases where histopathology showed a tumor at the lateral boundary of the excision (i.e., positive surgical margins), greater than one Mohs stage was required to completely remove the cancer. When this occurred, margin delineation results from imaging were considered true positive (TP) if collagen disruption extended beyond the surgeon’s marker in the same topographical area as in the intraoperative Mohs map. The OPI evaluation was categorized as false positive (FP) if imaging displayed collagen distortion past a clear surgical margin, as indicated by histopathology. Sensitivity and specificity of spectrally encoded OPI were determined using the following equations:

Sensitivity = TP/(TP + FN)(2)

Specificity = TN/(TN + FP)(3)

## 3. Results

### 3.1. System Characterization 

A handheld optical polarization imaging system was designed and built to enable rapid assessment of skin cancer tumor margins with minimal interruption to current treatment workflows. The newly designed imager has a mass of 0.55 kg (1.21 lbs.), dimensions of 10 cm × 10 cm × 22 cm, and a field of view (FOV) of 3.2 × 2.3 cm^2^. Lateral resolution was measured to be 12.4 µm (40.3 line pair/mm). Image acquisition times ranged between 5 ms and 50 ms. Power density of light incident on the skin was 0.2 mW/cm^2^ at 440 nm, 0.4 mW/cm^2^ at 570 nm, and 0.5 mW/cm^2^ at 640 nm. These values are lower than the maximum permitted skin/ocular exposures established by the American National Standards Institute (ANSI) [28].

Table 1 compares characteristics of the new imager with those of the previous prototype [29,30]. It demonstrates that the handheld OPI device is twice as light, its field of view is two times larger, while the lateral resolution remains the same. The image acquisition times are 10 times shorter, while the power densities are similar. In addition, it allows for imaging at 570 nm and incorporates automated image processing.

### 3.2. Clinical Validation

To validate the handheld OPI device, we imaged 10 subjects with pathologically diverse BCC lesions. Table 2 summarizes the clinical/experimental data and results. Subjects included seven men and three women between 55–83 years old (mean age: 71 ± 9 years). In total, 10 cancerous lesions were imaged and analyzed. Histopathological analysis revealed tumor subtypes of nodular BCC (*n* = 5), superficial BCC (*n* = 1), nodular/superficial BCC (*n* = 2), nodular BCC with squamous differentiation (*n* = 1), and micronodular/cystic BCC with ductal differentiation (*n* = 1). The skin cancers were located on the face (*n* = 7; left temple, right eyebrow, glabella, right nasal, right cheek, left cheek, and right jaw) or on the upper/lower extremity (*n* = 3; right shoulder, right forearm, and right tibia). Tumor dimensions ranged from 0.4 × 0.4 cm^2^ to 2.5 × 2.0 cm^2^ (mean size: 1.22 ± 0.57 cm^2^). Four of the cases (40%) required two stages of Mohs surgery to completely remove the cancer, whereas six cases (60%) needed one Mohs stage.

The results demonstrate that preoperative margin delineation using spectrally encoded OPI correlated with the findings of clinical histopathology in nine out of ten cases. Specifically, in all six cases with negative margins after the first Mohs excision, OPI correctly showed the tumor contained inside clinical borders marked by the surgeon (i.e., there were six TN cases). Four cases required >1 Mohs stage to clear the lesion because preoperative visual assessment underestimated the lateral tumor size (i.e., a 40% error rate from the surgeon). In three of the cases with positive margins, OPI accurately displayed the tumor extending beyond the surgical marker (i.e., three TP cases). In one case, OPI correctly detected the positive margin, but incorrectly represented blood vessels as cancer in another region (i.e., one FP case). Based on results of the pilot study, handheld OPI demonstrated a sensitivity of 100% and specificity of 86%.

**Figure 2:**
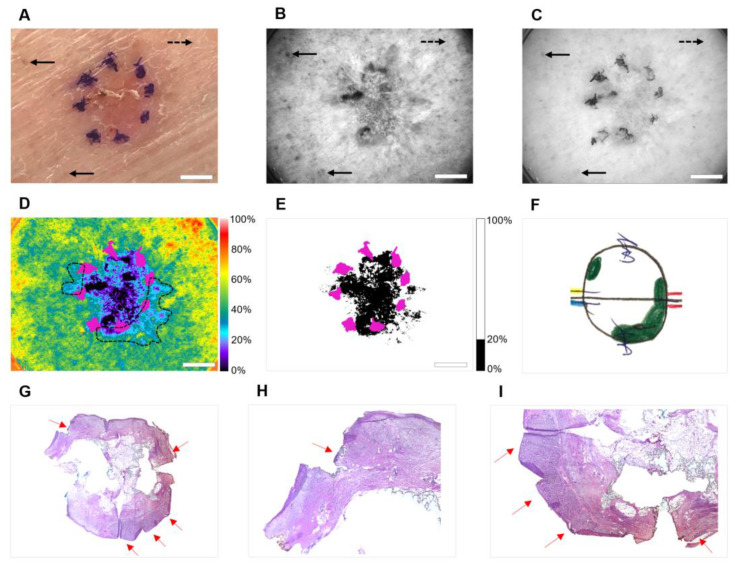
Example case with positive surgical margins (subject #9). (**A**) Preoperative photograph of BCC lesion outlined with surgical marker; (**B**) 440 nm cross-polarized image; (**C**) 640 nm cross-polarized image; (**D**) Pseudo-colored, spectrally encoded image overlaid with surgical marker (shown in magenta). Positive margins have been outlined in dashed black lines. (**E**) Binary, spectrally encoded image overlaid with surgical marker (threshold = 20%); (**F**) Intraoperative Mohs map (positive margins are drawn in green); (**G**) H&E histology; (**H**) Magnified view of positive surgical margin (10:00 o’clock position) in histology section; (**I**) Magnified view of positive surgical margin (3:00–6:00 o’clock position) in histology section (rotated 90°). The color bars in (**D**,**E**) show pixel values as a percentage of the maximum pixel value in the image. Solid black arrow: pigmentation spot; dashed black arrow: blood vessel; solid red arrow: residual tumor at lateral boundary of H&E section. Scale Bar = 5 mm.

To demonstrate the clinical performance of handheld OPI, in Figure 2 we present example images of the 83-year-old female patient with basal cell carcinoma (subject # 9) that required two stages of Mohs surgery. A preoperative photograph of the tumor outlined with a surgical marker (Figure 2A) exhibits limited visual contrast between cancerous and normal skin. Figure 2B shows the gray-scale optical image acquired at 440 nm. Reduced reflectance signal caused by collagen disruption, pigmented macules, and blood vessels is visible. Figure 2C shows the lesion imaged at 640 nm where pigmentation spots, blood vessels, and surgical markings can be seen. The pseudo-colored (Figure 2D) and binary (Figure 2E) spectrally encoded images were processed using Equation (1). Processed images were superimposed with the surgeon’s marks. The images (Figure 2D,E) demonstrate the significantly decreased impact of background melanin and hemoglobin. Two positive margins at the left and right borders of the excision (10:00 o’clock and 3:00–6:00 o’clock positions, respectively) correlate well with the Mohs map (Figure 2F). Clinical H&E histopathology (Figure 2G–I) confirmed the diagnosis of nodular BCC with squamous differentiation.

## 4. Discussion

OPI technology identifies tumor-induced disruption of the dermal collagen structure to map lateral margins of skin cancer [29,30]. Previously, a pilot trial of six cases showed that noninvasive collagen assessment by OPI provides a reliable biomarker for skin cancer [29]. More recently, in a clinical study of 53 cases conducted at Massachusetts General Hospital, we demonstrated that spectrally encoded OPI accurately detects NMSC borders by minimizing the impact of skin chromophores (e.g., melanin and hemoglobin) [30]. However, a previous prototype of the imaging system was not practical in the context of fast-paced clinical applications, as it was bulky, heavy, required an articulating arm for positioning, and relied on manual data processing. The new handheld imager (Figure 1B) is compact, lightweight, and easy to operate. The designed and implemented handheld OPI system is capable of in vivo delineation of BCC lesions within seconds, and therefore it does not disrupt current treatment workflows. The technology requires only minimal training to operate the device and interpret the images. The spectral encoding algorithm was developed and programmed to detect tumor boundaries in biopsy-proven BCC in conjunction with available clinical information (e.g., visible features of the lesion). Automated data processing further decreases requirements for operator performance. The handheld device was validated by imaging 10 cases. Studies including larger case numbers are underway to establish its diagnostic accuracy in clinical practice.

Other optical techniques are being developed for skin cancer margin delineation. For example, dermoscopy typically utilizes non-polarized, white light illumination to visualize the skin surface. However, studies have indicated that this technique does not enhance the accuracy of the preoperative tumor margin delineation [31,32]. Recently, RCM and OCT modalities have been combined into a single device for NMSC margin assessment [24,25,26]. These systems offer cellular level resolution of RCM (axial resolution: 1–3 μm; lateral resolution: 0.5–1.0 μm) with 1–2 mm imaging depth of OCT. However, a small FOV (RCM mode: <1 mm; OCT mode: ~2 mm) presents a significant challenge. Single images can be stitched together to visualize large areas. However, mosaicking is time-consuming, sensitive to motion artifacts, and uses sophisticated software. The available RCM/OCT systems are expensive, and image acquisition/interpretation requires extensive training. Moreover, the imaging results are based on the subjective evaluation of tissue morphology. In comparison, spectrally encoded OPI with pseudo-color provides a wide FOV and displays the tumor as a black and purple region surrounded by highly reflective collagen (Figure 2D).

**Figure 3:**
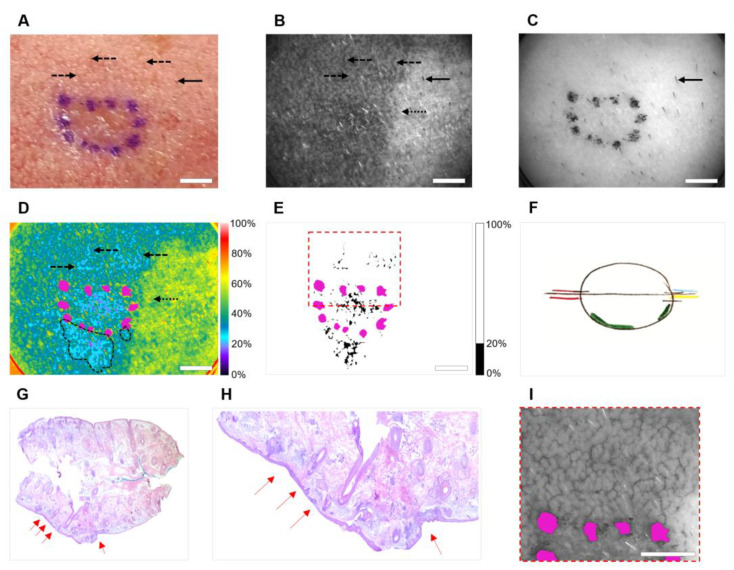
Case with false positive OPI margin assessment (subject #6). (**A**) Preoperative photograph of BCC lesion outlined with surgical marker; (**B**) 440 nm cross-polarized image; (**C**) 640 nm cross-polarized image; (**D**) Pseudo-colored, spectrally encoded image overlaid with surgical marker (shown in magenta). Positive margins have been outlined in dashed black lines. (**E**) Binary, spectrally encoded image overlaid with surgical marker (threshold = 20%); (**F**) Intraoperative Mohs map (positive margins are drawn in green); (**G**) H&E histology; (**H**) Magnified view of positive surgical margins in histology section; (**I**) Magnified view of region outlined by dashed red lines in (**E**) imaged at 570 nm. The color bars in (**D**,**E**) show pixel values as a percentage of the maximum pixel value in the image. Dashed black arrow: blood vessel; solid black arrow: hair follicle; dotted black arrow: nonuniform illumination; solid red arrow: residual tumor at lateral boundary of H&E section. Scale Bar = 5 mm.

Images of a 55-year-old male patient with superficial basal cell carcinoma on the left cheek are shown in Figure 3. This was the only false positive case imaged in this study, where OPI accurately detected two positive margins, but misinterpreted blood vessels as a tumor in an upper part of the image. The preoperative photograph (Figure 3A) shows the borders of the superficial BCC as estimated by the surgeon. Prominent vasculature surrounding the lesion is clearly visible. Figure 3B,C shows optical images of the lesion acquired at 440 nm and 640 nm, respectively. The 440 nm image (Figure 3B) visualizes collagen, hemoglobin, and hair follicles. The 640 nm image (Figure 3C) shows hair and the surgeon’s marker. A comparison of pseudo-colored (Figure 3D) and black/white (Figure 3E) spectrally encoded images with the Mohs map (Figure 3F) and clinical histology (Figure 3G,H) reveals that OPI correctly identified an extension of the tumor beyond surgical marks at two locations on the lower margin of the excision and misidentified blood as cancer above the upper margin. Figure 3I presents a magnified view of the region outlined by a red dashed line in Figure 3E, imaged at 570 nm. The wavelength of 570 nm corresponds to the absorption maximum of hemoglobin; therefore, Figure 3I visualizes numerous blood vessels present at the upper border of the lesion.

In this case, suboptimal imaging conditions and prominent superficial vasculature contributed to the false positive assessment by OPI. Nonuniform pressure on the lesion caused uneven illumination across the FOV (Figure 3B–D). Applying moderate, consistent pressure would have eliminated the illumination gradient and could have mitigated the impact of hemoglobin by temporarily pushing blood out of the papillary dermis vessels. OPI image acquisition takes less than one second and the frames can be viewed in real time. Therefore, the operator could be trained to recognize these potential issues, reposition the device as needed, and repeat clinical imaging. In addition, a spectral encoding algorithm incorporating 570 nm image, which emphasizes blood vessels (Figure 3I), could further minimize the impact of hemoglobin.

## 5. Conclusions

This work demonstrated the feasibility of the first handheld OPI prototype for rapid, noninvasive preoperative delineation of BCC margins. The device is capable of wide-field (~3 × 2 cm^2^) rapid image acquisition (~5–50 ms) with lateral resolution of ~12 µm. It is compact and lightweight (0.55 kg). Clinical evaluation of the prototype has shown its superior performance compared to the surgeons’ visual preoperative assessment of lateral cancer margins. Overall, the presented results point towards the significant potential of optical polarization collagen imaging for guiding skin cancer treatments and improving their outcomes without altering clinical workflows. Future research directions include further miniaturization of the technology and exploring its potential for cancer screening and guiding treatments other than surgery.

## Figures and Tables

**Table 1:** Comparison of handheld OPI with previous imaging system.

Specifications		Handheld OPI	Previous System
Weight		1.21 lbs.	2.51 lbs.
Field of View		3.2 × 2.3 cm^2^	2.2 × 1.6 cm^2^
Lateral Resolution		12.4 µm	12.4 µm
Image Acquisition Time	440 nm	50 ms	620 ms
	570 nm	41 ms	n/a
	640 nm	5 ms	64 ms
Power Density	440 nm	0.2 mW/cm^2^	0.2 mW/cm^2^
	570 nm	0.4 mW/cm^2^	n/a
	640 nm	0.5 mW/cm^2^	0.6 mW/cm^2^
Data Processing		Automated	Manual

n/a, not applicable.

**Table 2:** Clinical information and experimental data for 10 study subjects.

Subject No.	Sex	Age	Diagnosis	Tumor Site	Tumor Size [cm]	No. of Mohs Stages	OPI Classification	OPI/Histopathology Correlation
1	M	76	BCC, nodular	Glabella	0.4 × 0.4	1	TN	+
2	M	71	BCC, nodular	R forearm	1.7 × 1.2	1	TN	+
3	M	77	BCC, nodular	R cheek	1.1 × 0.7	1	TN	+
4	M	67	BCC, nodular	L temple	1.0 × 0.8	1	TN	+
5	F	81	BCC, nodular	R nasal	1.1 × 0.6	2	TP	*+*
6	M	55	BCC, superficial	L cheek	1.0 × 0.5	2	FP *	+/−
7	M	69	BCC, nodular/superficial	R jaw	1.1 × 0.9	1	TN	+
8	F	72	BCC, nodular/superficial	R eyebrow	0.6 × 0.6	2	TP	+
9	F	83	BCC, nodular with squamous differentiation	R tibia	1.7 × 1.1	2	TP	+
10	M	60	BCC, micronodular/cystic with ductal differentiation	R shoulder	2.5 × 2.0	1	TN	+

R, right; L, left; BCC, basal cell carcinoma; TN, true negative; TP, true positive; FP, false positive; +, case where OPI assessment correlated with histological findings; +/−, case where OPI assessment partially correlated with histological findings; *, this case was judged FP because OPI correctly identified positive margins, but misidentified blood vessels as tumor.

## Data Availability

The experimental data are available from the corresponding author upon request.
